# SOX9-activated *FARSA-AS1* predetermines cell growth, stemness, and metastasis in colorectal cancer through upregulating *FARSA* and *SOX9*

**DOI:** 10.1038/s41419-020-03273-4

**Published:** 2020-12-14

**Authors:** Taicheng Zhou, Lili Wu, Ning Ma, Fuxin Tang, Zhuomin Yu, Zhipeng Jiang, Yingru Li, Zhen Zong, Kunpeng Hu

**Affiliations:** 1 Department of Gastroenterological Surgery and Hernia Center, The Sixth Affiliated Hospital of Sun Yat-sen University, Guangdong Institute of Gastroenterology, Guangdong Provincial Key Laboratory of Colorectal and Pelvic Floor Diseases, Supported by National Key Clinical Discipline, 510655 Guangzhou, Guangdong China; 2 Department of Medical Ultrasonics, Third Affiliated Hospital of Sun Yat-sen University, Guangdong Key Laboratory of Liver Disease Research, 510630 Guangzhou, Guangdong China; 3 Department of Gastroenterological Surgery, The Second Affiliated Hospital of Nanchang University, No.1 Mingde Road, 330006 Nanchang, Jiangxi China; 4 Department of General Surgery, The Third Affiliated Hospital of Sun Yat-sen University, No.2693 Kaichuang Road, Huangpu, 510000 Guangzhou, Guangdong China

**Keywords:** Colorectal cancer, Cell biology

## Abstract

SRY-box transcription factors (SOXs) are effective inducers for the formation of stem-like phenotypes. As a member of SOX family, *SOX9* (SRY-box transcription factor 9) has been reported to be highly expressed and exert oncogenic functions in multiple human cancers. In this study, we hypothesized that *SOX9* could regulate the function of cancer stem/initiating cells (CSCs) to further facilitate the progression of colorectal cancer (CRC). Then, stable transfection of shRNAs was used to silence indicated genes. Loss-of-function experiments were conducted to demonstrate the in vitro function of CRC cells. In vivo study was conducted to determine the changes in tumorigenesis and metastasis in vivo. Bioinformatics analyses and mechanistic experiments were employed to explore the downstream molecules. Presently, GEPIA data indicated that *SOX9* was upregulated in 275 COAD (colon adenocarcinoma) samples relative to 349 normal tissues. Besides, we also proved the upregulation of *SOX9* in CRC cell lines (HCT15, SW480, SW1116, and HT-29) compared to normal NCM-460 cells. Silencing of *SOX9* suppressed cell growth, stemness, migration, and invasion. Mechanistically, SOX9 activated the transcription of lncRNA phenylalanyl-tRNA synthetase subunit alpha antisense RNA 1 (*FARSA-AS1*), while *FARSA-AS1* elevated *SOX9* in turn by absorbing *miR-18b-5p* and augmented *FARSA* via sequestering *miR-28-5p*. Furthermore, loss of *FARSA-AS1* hindered malignant phenotypes in vitro and blocked tumor growth and metastasis in vivo. Notably, we testified that *FARSA-AS1* aggravated the malignancy in CRC by enhancing *SOX9* and *FARSA*. Our study unveiled a mechanism of SOX9-*FARSA-AS1*-*SOX9*/*FARSA* loop in CRC, which provides some clews of promising targets for CRC.

## Introduction

Colorectal cancer (CRC) has been reported as one of the most malignant cancers in the world^1,2^. Because of the high occurrence and death rate of CRC, people’s health has been affected strictly. Even though there are much advance in the therapy of CRC, CRC still acts as a serious disease. It has been studied that various oncogenes participate in the progression of CRC, which provides novel targets for treating CRC^3^. Nevertheless, it is still unclear about the exact function and mechanism of oncogenes played in CRC.

As a member of SOX family, SRY-box transcription factor 9 (SOX9) can regulate cancer progression by affecting the transcription of diverse genes^4,5^. It has been reported that *SOX9* is involved in the progression of prostate cancer and gastric cancer by regulating WNT signaling pathway^6,7^. Meanwhile, *SOX9* can modulate cell stemness in hepatocellular carcinoma^8,9^. Also, inhibited *SOX9* hampers cell growth, migration, invasion, and EMT in thyroid cancer^10^. *SOX9* can regulate the self-renewal of cancer stem cells in hepatocellular carcinoma^11^. However, the function of *SOX9* in CRC still remains largely unknown. In this study, we are going to search the role and probable molecular mechanism of *SOX9* in CRC.

With the development in sequencing technologies, it has been clearer that long noncoding RNAs (lncRNAs) are a novel group of RNA transcripts with more than 200 nucleotides in length^12^. More and more studies have proved that lncRNAs are dysregulated in cancers, and lncRNAs can participate in the regulation on biological behaviors of cancer cells, such as cell growth, apoptosis, migration, and invasion^13,14^. For example, lncRNA *NEAT1* affects cell growth and apoptosis in CRC^15^. Also, lncRNA *AB073614* regulates cell growth and metastasis by modulating PI3K/AKT pathway in CRC^16^. Besides, lncRNA *TUG1* regulates the chemoresistance of CRC cells^17^. Phenylalanyl-tRNA synthetase subunit alpha antisense RNA 1 (*FARSA-AS1*) is a newly found lncRNA that has been scarcely explored in CRC. In our study, we are going to explore the function and underlying molecular mechanism of *FARSA-AS1* in CRC.

The competing endogenous RNA (ceRNA) network has recently been proposed, in which lncRNAs can function as microRNAs (miRNAs) sponges to regulate the expression of messenger RNAs (mRNAs) targeted by above miRNAs^18,19^. For example, lncRNA *NORAD* can function as a ceRNA to regulate EMT process and metastasis in pancreatic cancer^20^. Meanwhile, lncRNA *H19* can play the role of ceRNA to modulate EMT process and metastasis in bladder cancer via sponging *miR-29b-3p*^21^. Also, lncRNA *SNHG3* can be a ceRNA to promote the progression of CRC^22^. In our study, we searched the position of *FARSA-AS1* in CRC cells and further explored the possible molecular mechanism of *FARSA-AS1* in CRC.

## Materials and methods

### Cell lines and culture

Human CRC cell lines (HCT15, SW480, SW1116, and HT-29) and normal human colon epithelial cell line (NCM-460), all from ATCC (Manassas, VA), were allowed to propagate in the humidified incubator of 5% CO_2_ at 37 °C. DMEM medium (Gibco, Carlsbad, CA) was applied for culturing cells, with 1% Pen/Strep solution and 10% FBS (Gibco) as the supplements.

### Total RNA extraction and qRT-PCR

Total cellular RNAs were extracted using Trizol based on the standard protocol. After that, the obtained RNAs were then used for cDNA synthesis with PrimeScript RT reagent kit (Takara, Otsu, Japan). Gene expression was quantified by qRT-PCR using Power SYBR Green (TaKaRa) on ABI Prism 7900HT (Applied Biosystems, Foster City, CA, USA) and then calculated with 2^−ΔΔCt^ method. *GAPDH* or *U6* acted as the internal reference for normalization. The primer sequences were shown in Table 1.

**Table 1:** The sequences of PCR primers.

Genes	Forward primer (5’ > 3’)	Reverse primer (5’ > 3’)
*SOX9*	TCTGAACGAGAGCGAGAAGC	CCGTTCTTCACCGACTTCCT
*NOS2*	CGCATGACCTTGGTGTTTGG	GCACATCCCCGCAAACATAG
*FOS*	CAAGCGGAGACAGACCAACT	GTGAGCTGCCAGGATGAACT
*PVRL1 (NECTIN1)*	ATTCCCCTACACCCCGTCTC	GGGGTACTGCAGGTTCTGTG
*FARSA-AS1*	TCCTGCTATCGCTTCCCAGT	GGTTGCGACGTAATAGGAAGGT
*GAPDH*	GGAGCGAGATCCCTCCAAAAT	GGCTGTTGTCATACTTCTCATGG
*U6*	CTCGCTTCGGCAGCACA	AACGCTTCACGAATTTGCGT
*FARSA*	GCCCTTCAAGCCCTACAACT	CTGGAAGAGGGCGTCAAAGT
*ALDH1A1*	CTGCCGGGAAAAGCAATCTG	TCTTAGCCCGCTCAACACTC
*CD133*	GTGGCGTGTGCGGCTATGAC	CCAACTCCAACCATGAGGAAGACG
*miR-18a-5p*	GCGAGTAAGGTGCATCTAGT	CTCAACTGGTGTCGTGGA
*miR-18b-5p*	GCGAGTAAGGTGCATCTAGT	CTCAACTGGTGTCGTGGA
*miR-28-5p*	CGAGAAGGAGCTCACAGTCT	CTCAACTGGTGTCGTGGA
*miR-708-5p*	GCGAGAAGGAGCTTACAATC	CTCAACTGGTGTCGTGGA

### Western blot

Based on the instruction, total cellular protein extracts were acquired with RIPA lysis buffer, dissolved with 10% SDS-PAGE and then transferred to PVDF membranes. Followed by sealing with 5% skim milk, membranes were processed with primary antibodies against GAPDH (loading control) SOX9, and FARSA, and then with the secondary antibodies tagged with HRP subsequently. The antibodies were all purchased from Abcam (Cambridge, MA) and employed after dilution. Protein bands were visualized with the help of the enhanced chemiluminescence (ECL) fluorescence test kit (Amersham, Arlington Heights, IL).

### Transfection

The specifically designed shRNAs and NC shRNAs were produced by Genepharma Company (Shanghai, China) for the depletion of *SOX9* and *FARSA-AS1* in SW480 and SW1116 cells. Besides, the pcDNA3.1-SOX9 and pcDNA3.1-FARSA were obtained through inserting corresponding cDNA sequences into pcDNA3.1 vectors (Invitrogen) for overexpressing *SOX9* and *FARSA*, with the empty vector as negative controls. MiR-18b-5p/miR-28-5p mimics and corresponding NC mimics (Genepharma) were constructed for overexpressing *miR-18b-5p* or *miR-28-5p*, respectively. Cell transfection was achieved using Lipofectamine 2000 (Invitrogen, Carlsbad, CA). Samples were collected after 48 h of transfection.

### Colony formation assay

The cultured cells at logarithmic growth were harvested and added to the six-well plates for the 14-day culture process at 37 °C in 5% CO_2_. After washing by PBS and fixing with 4% paraformaldehyde, cells were processed with crystal violet solution for staining, followed by counting manually.

### EdU assay

Cultured cells were placed at 5 × 10^4^ cells/well to the 96-well plates for treating with EdU staining kit (Ribobio, Guangzhou, China). After that, cell nuclei were dual-stained with DAPI solution, and then cells were subjected to final observation with fluorescence microscope (Olympus, Tokyo, Japan).

### Flow cytometer analysis of cell apoptosis

On the basis of protocol, cell apoptosis was monitored with Annexin-V fluorescein isothiocyanate (FITC)/propidium iodide (PI) dual-staining kit (BD Biosciences, San Jose, CA). In brief, after treating with FITC-Annexin-V and PI in succession for 15 min in the dark, the apoptotic cells were analyzed using FACS cytometry (BD Biosciences).

### Sphere-formation assay and secondary and tertiary tumor sphere-formation assays

SW480 and SW1116 cells were plated in 96-well ultralow attachment plates (Corning, New York, NY) and cultured in the sphere medium of DMEM with 10 ng/mL EGF (Peprotech Inc., Rocky Hill, NJ, USA), 10 ng/mL bFGF (Peprotech Inc., Rocky Hill, NJ, USA), and 1×B27 (Invitrogen). After 1 week, the number of spheres was counted and the images were taken under an optical microscope (Olympus). In addition, the secondary and tertiary tumor sphere-formation assays were conducted according to the previous protocol^23^.

### Transwell assay

The upper side of polycarbonate transwell filter (Corning) chamber was coated with Matrigel (BD Biosciences) for invasion assay and the chambers without Matrigel were prepared for migration analysis. Then, to the upper chambers were added with 5 × 10^4^ cells in serum-free medium, while the complete medium in the lower chamber acted as the supplements. One day later, the migrating or invading cells in the bottom were processed with 0.5% crystal violet after fixing by 4% paraformaldehyde, followed by cell counting under the microscope.

### Luciferase reporter assays

The fragments of FARSA-AS1 promoter with wild-type or mutant SOX9 binding sites were inserted into pGL3-basic vectors (Promega, Madison, WI). Then the recombinant constructs were subjected to the cotransfection with *SOX9* silencing plasmids into CRC cells. The pmirGLO-FARSA-AS1-WT/Mut and pmirGLO-SOX9 3’UTR-WT/Mut reporters were formed using the corresponding full-length sequences with wild-type or mutated *miR-18b-5p* binding sites, or pmirGLO-FARSA 3’UTR reporter with full-length FARSA 3’UTR sequence, were constructed and then cotransfected severally with miR-18b-5p mimics or NC mimics into CRC cells. Similarly, the pmirGLO-FARSA-AS1-WT/Mut and pmirGLO-FARSA-WT/Mut reporters were obtained through cloning corresponding full-length sequences covering wild-type or mutant *miR-28-5p* binding sites into pmirGLO vectors (Promega), and then were applied for cotransfecting with miR-28-5p mimics or NC mimics into CRC cells. The activities of firefly luciferase and Renilla luciferase were both tested by using dual luciferase reporter gene assay kit (BioTek, Winooski, VT) to determine the relative luciferase activity.

### TOP/FOP-flash assay

TOP/FOP-flash assay was carried out as described previously^24^. In short, cells were cotransfected with TOP/FOP-Flash (Genechem) and different plasmids for *SOX9* overexpression, *SOX9* inhibition, or *FARSA-AS1* inhibition. After normalizing to the Renilla luciferase activity, the TOP/FOP ratio was measured via dual luciferase reporter system (Promega).

### ChIP assay

One percent formalin was used to fix CRC cells to obtain DNA-protein cross-linking, followed by the cutting of DNA fragments into 200–500 bp via ultrasonic. Cell lysates with indicated fragments were subjected to immunoprecipitation with SOX9 antibody or control IgG antibody (Millipore, Billerica, MA). Then the precipitated DNA fragments were captured by using magnetic beads. The qRT-PCR was employed for quantifying the precipitated DNA.

### Pull-down analyses

For DNA pull-down, the 5’ biotin-labeled FARSA-AS1 promoter and non-labeled promoter probes were conjugated with beads for culturing the protein extracts from CRC cells for 2 h. Proteins were subjected to western blot analysis. For RNA pull-down, the wild-type and mutated *miR-18b-5p* fragments covering *FARSA-AS1* or *SOX9* interacting sequences were synthesized and biotinylated into Bio-miR-18b-5p-WT/Mut probes. The wild-type and mutated *miR-28-5p* fragments containing *FARSA-AS1* or *FARSA* interacting sequences were acquired and biotinylated into Bio-miR-28-5p-WT/Mut probes. After incubation with cell extracts, qRT-PCR was carried out to detect indicated RNAs.

### FISH

The specific RNA FISH probe of *FARSA-AS1* was synthesized by Bersinbio Company (Guangzhou, China) and applied for FISH assay as required by supplier. After DAPI dual-staining, cells were imaged under fluorescence microscope.

### IF staining

To detect SOX9 localization in CRC cells, IF assay was implemented with specific SOX9 antibody purchased from Abcam. Simply put, cells were fixed with 4% paraformaldehyde for 10 min, permeabilized via 0.1% Triton X-100 for 5 min and then sealed with 1% BSA for 1 h. Afterwards, cells were then incubated overnight with SOX9 antibodies at 4 °C, followed by the incubation for 1 h with the AlexaFluor® 488 secondary antibodies at room temperature. Following staining via DAPI, cells were observed under a fluorescence microscope.

### Subcellular fractionation

The separation of cell nucleus and cytoplasm was achieved with the PARIS Kit (Invitrogen) on the basis of standard method. Then, the level of indicated RNAs (*FARSA-AS1*, *GAPDH*, or *U6*) in different fractions was analyzed via qRT-PCR, with *GAPDH* and *U6* acted as cytoplasmic and nuclear indicators, respectively.

### Animal experiment

Six weeks old of female BALB/c nude mice (SLRC Laboratory Animal Center, Shanghai, China) were commercially acquired for animal study, with the approval of the Institutional Animal Care and Use Committee of the Sixth Affiliated Hospital of Sun Yat-sen University. Each mouse was injected subcutaneously with 3 × 10^6^ CRC cells that were transfected with sh-NC or sh-FARSA-AS1 (three mice each group), with tumor volume measured every 4 days. Four weeks later, mice were sacrificed, and then the xenografts were collected for weight mensuration and qRT-PCR analysis. For in vivo metastasis analysis, 1 × 10^7^ CRC cells transfected with sh-NC or sh-FARSA-AS1 were injected into the tail vein of mice. Six weeks later, the lungs of mice (total three lungs each group) were collected for counting the pulmonary metastatic nodules. The lungs fixed in formalin were processed with paraffin embedding for Hematoxylin and Eosin (H&E) staining.

### Statistical analysis

Data from independent bio-triplications were shown as mean ± standard deviation (SD) and analyzed via Graphpad Prism 6 software. The comparison of differences between groups was appropriately achieved by *t*-test (two-tailed) and one-way or two-way ANOVA, as per the significant level at *p* < 0.05.

## Results

### SOX9 is upregulated in CRC cells and promotes cell growth, stemness, migration, and invasion

**Fig. 1:**
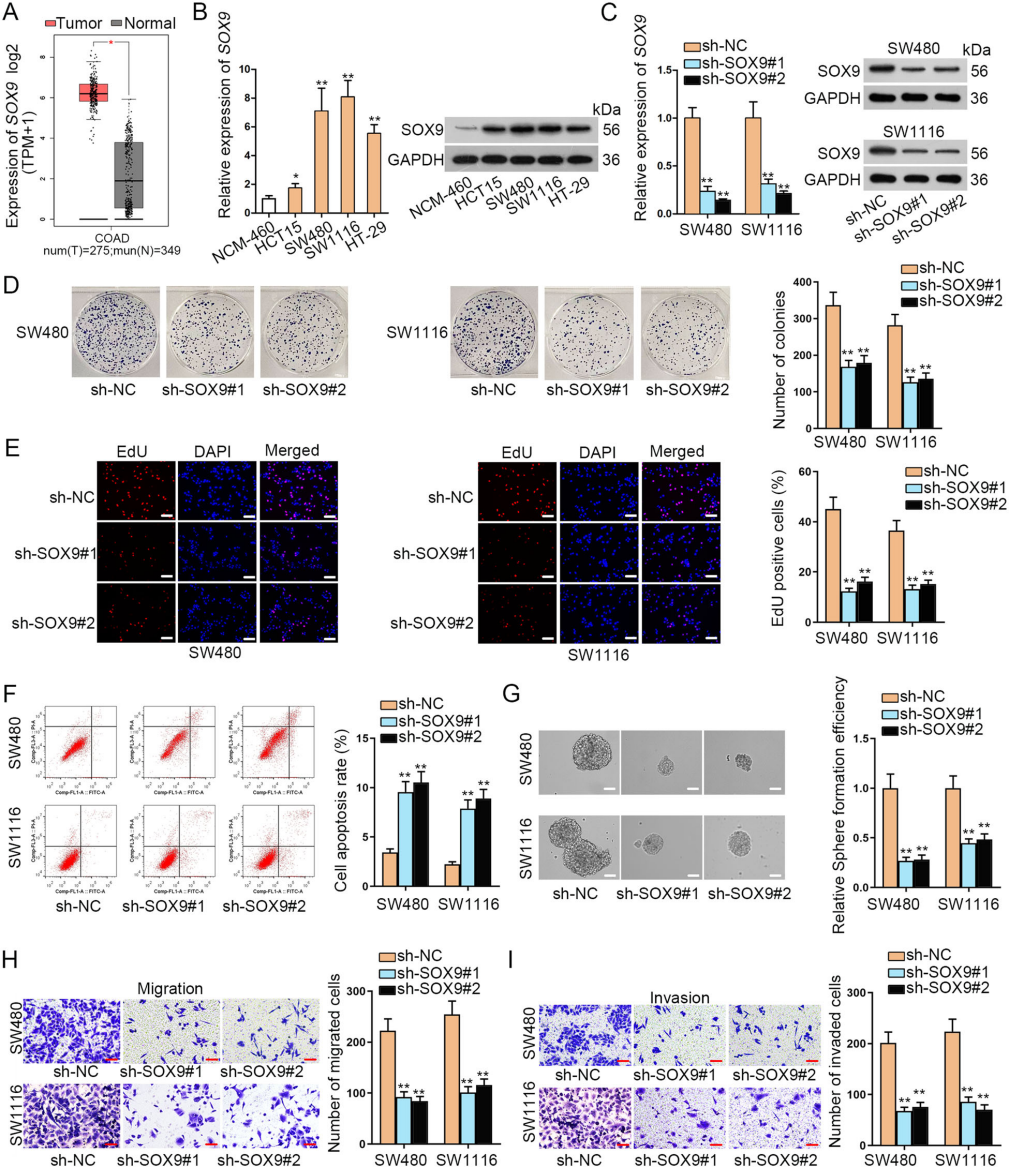
*SOX9* is upregulated in CRC cells and promotes cell growth, stemness, migration, and invasion. **A** GEPIA data showed the expression of *SOX9* in 275 COAD (colon adenocarcinoma) samples and 349 normal colon tissues. **B** The qRT-PCR and western blot experiments were utilized to detect the expression of *SOX9* in CRC cell lines and normal NCM-460 cells. **C** The knockdown efficiency of *SOX9* in SW480 and SW1116 cells was tested by qRT-PCR and western blot experiments. **D, E** The proliferation capability of cells was detected by colony formation assay and EdU assay (scale bar = 150 μm). **F** Flow cytometry was utilized to test the apoptotic cells marked with FITC-Annexin-V or together with FITC-Annexin-V and PI. **G** Tumor sphere-formation assay was utilized to detect cell stemness (scale bar = 75 μm). **H, I** Transwell assay was adopted to test cell migration and invasion under different contexts (scale bar = 250 μm). *P < 0.05, **P < 0.01.

According to the data from GEPIA (http://gepia.cancer-pku.cn/), we found that *SOX9* was upregulated in colon adenocarcinoma (COAD) tissues (Fig. 1A). Thereafter, we planned to detect the expression of *SOX9* in CRC cell lines (HCT15, SW480, SW1116, and HT-29) and human normal colonic epithelial cell (NCM-460). Before that, we identified that no *SOX9* mutation was found in SW1116 and HT-29 cells but four kinds of *SOX9* mutations were predicted in HCT15 cells by using COSMIC tool (http://cancer.sanger.ac.uk/cancergenome/projects/cell_lines/). Besides, a previous study also suggested no *SOX9* mutation in SW480 cell line^25^. Then the results of qRT-PCR showed the high levels of SOX9 mRNA and protein in three CRC cell lines with wild-type *SOX9*, especially in SW480 and SW1116 cells (Fig. 1B). Afterwards, we knocked down *SOX9* expression in SW480 and SW1116 cells for further study (Fig. 1C). Data from colony formation assay and EdU assay indicated that loss of *SOX9* inhibited the proliferative capacity of SW480 and SW1116 cells (Fig. 1D, E). In contrast, the apoptosis of SW480 and SW1116 cells was induced by *SOX9* silencing (Fig. 1F). Importantly, we found that the efficiency of sphere formation by SW480 and SW1116 cells was restrained and the size of spheres was diminished under the inhibition of *SOX9* (Fig. 1G). To further explore the impact of *SOX9* on CRC cell stemness, we conducted secondary and tertiary tumor sphere-formation assays. Results manifested that the absence of *SOX9* hindered the formation of secondary and tertiary tumor spheres from CRC cells (Fig. S1A). Consistently, we also discovered the reduced levels of *ALDH* and *CD133* in CRC cells with *SOX9* depletion (Fig. S1B). Furthermore, the outcomes of transwell assay unveiled that the migration and invasion capacities were also hampered in *SOX9* silenced SW480 and SW1116 cells (Fig. 1H, I). To further know the influence of *SOX9* on the malignant behaviors of colorectal cells, we performed gain-of-function assays in the normal NCM-460 cells. It was proved that upregulating *SOX9* in NCM-460 cells facilitated proliferation, enhanced stemness and accelerated migration and invasion (Fig. S1C-I). Besides, considering the importance of *SOX9* in regulating Wnt/β-catenin pathway, we then tested its impact on this pathway in CRC. As expected, *SOX9* deficiency dramatically lowered the activity of Wnt/β-catenin signaling in SW480 and SW1116 cells, whereas its overexpression evidently fortified the activity of this pathway in NCM-460 cells (Fig. S1J). These data suggested that *SOX9* is upregulated in CRC cells and it promotes oncogenic phenotypes in CRC cells by activating Wnt/β-catenin pathway.

### SOX9 binds to FARSA-AS1 promoter to elevate FARSA-AS1 in CRC cells

**Fig. 2:**
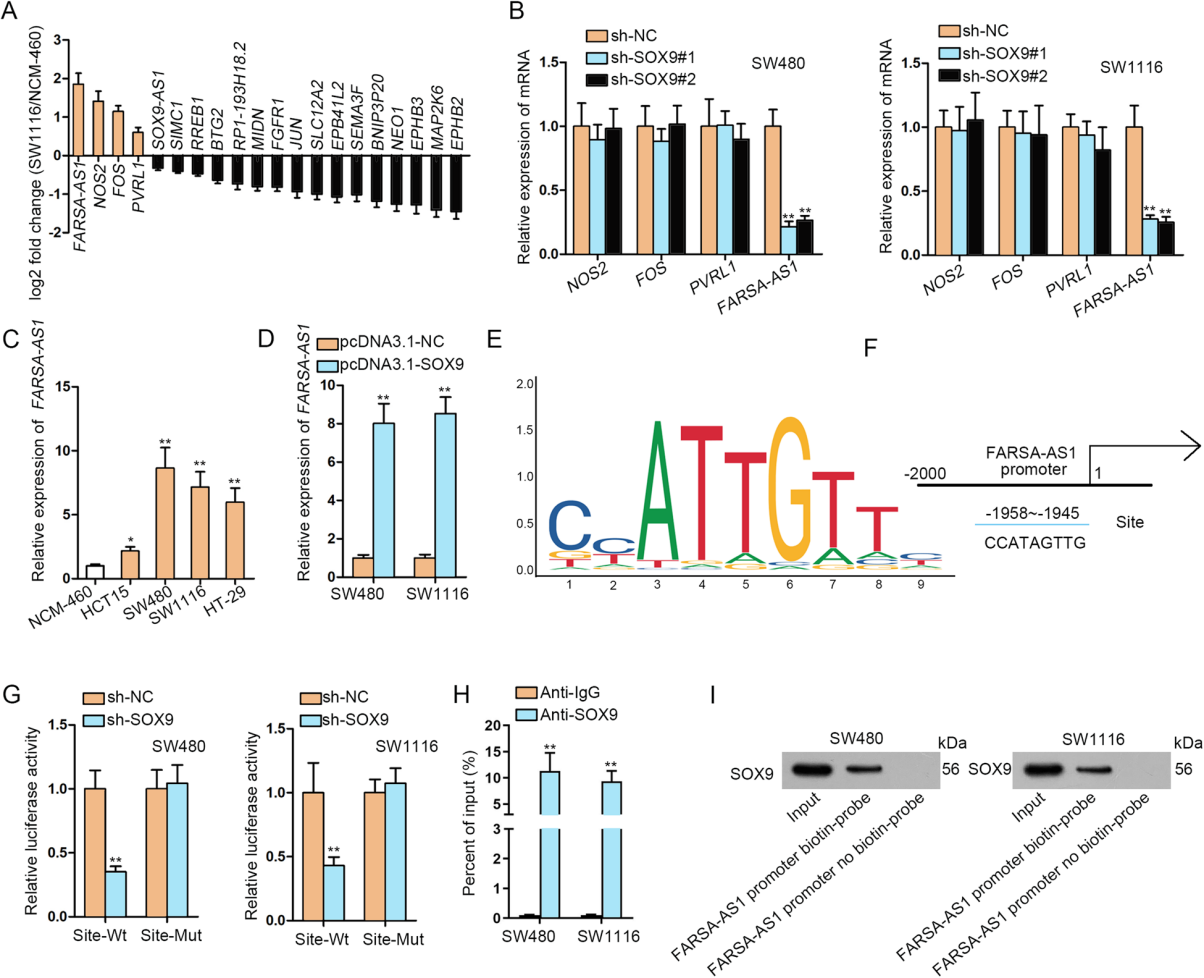
SOX9 binds to FARSA-AS1 promoter. **A** The expression of top 20 genes correlated with *SOX9* in COAD samples from GEPIA database was detected by qRT-PCR. **B** The qRT-PCR was adopted to test the effects of silencing *SOX9* on the expression of *NOS2*, *FOS*, *PVRL1*, and *FARSA-AS1*. **C** *FARSA-AS1* expression in CRC cells was tested by qRT-PCR. **D** The effect of overexpressing *SOX9* on the expression of *FARSA-AS1* in SW480 and SW1116 cells was detected by qRT-PCR. **E** The DNA motif for SOX9 was obtained from JASPAR. **F** SOX9 binding sites in FARSA-AS1 promoter were predicted by JASPAR. **G** Luciferase reporter assay was utilized to detect the combined situation of SOX9 in FARSA-AS1 promoter. **H, I** ChIP and DNA pull-down assays were adopted to prove the combination between SOX9 and FARSA-AS1 promoter. *P < 0.05, **P < 0.01.

Then, we researched the similar genes of *SOX9* in COAD samples from GEPIA (http://gepia.cancer-pku.cn/). As shown in Fig. 2A, top 20 genes correlated with *SOX9* in COAD samples were selected and the results of qRT-PCR indicated that four (*NOS2*, *FOS*, *PVRL1*, and *FARSA-AS1*) among the 20 genes presented upregulation in SW1116 cells compared to NCM-460 cells. Then, we detected the expression of these four genes in CRC cells with or without *SOX9* depletion, and results displayed that FARSA-AS1 was significantly downregulated in *SOX9* silenced SW480 and SW1116 cells (Fig. 2B). In addition, the expression of *FARSA-AS1* was found to be upregulated in CRC cells (Fig. 2C). Subsequently, we overexpressed *SOX9* in SW480 and SW1116 cells (Fig. S2A), resulting in the upregulation of *FARSA-AS1* in such cells (Fig. 2D). These data indicated that *SOX9* could positively regulated FARSA-AS1. It was reported that SOX9 could function as a transcription factor and activate gene expression by binding to the promoter of genes^26^. Here, we also discovered that a major proportion of SOX9 located in the nucleus of CRC cells (Fig. S2B), further highlighting the potential for SOX9 to affect FARSA-AS1 transcription. To confirm this, we used UCSC (http://genome.ucsc.edu/) and JASPAR (http://jaspar.genereg.net/) websites to obtain the DNA motif and the binding site of SOX9 on FARSA-AS1 promoter (Fig. 2E, F). Luciferase reporter assay results revealed that *SOX9* silencing decreased the luciferase activity of wild-type FARSA-AS1 promoter, whereas that of FARSA-AS1 promoter with mutant binding site displayed no significant changes (Fig. 2G). Data from ChIP and DNA pull-down assays further confirmed the interactivity of SOX9 with FARSA-AS1 promoter (Fig. 2H, I). Conclusively, *SOX9*transcriptionally activates *FARSA-AS1* in CRC cells.

### FARSA-AS1 facilitates the malignant processes in CRC cells

**Fig. 3:**
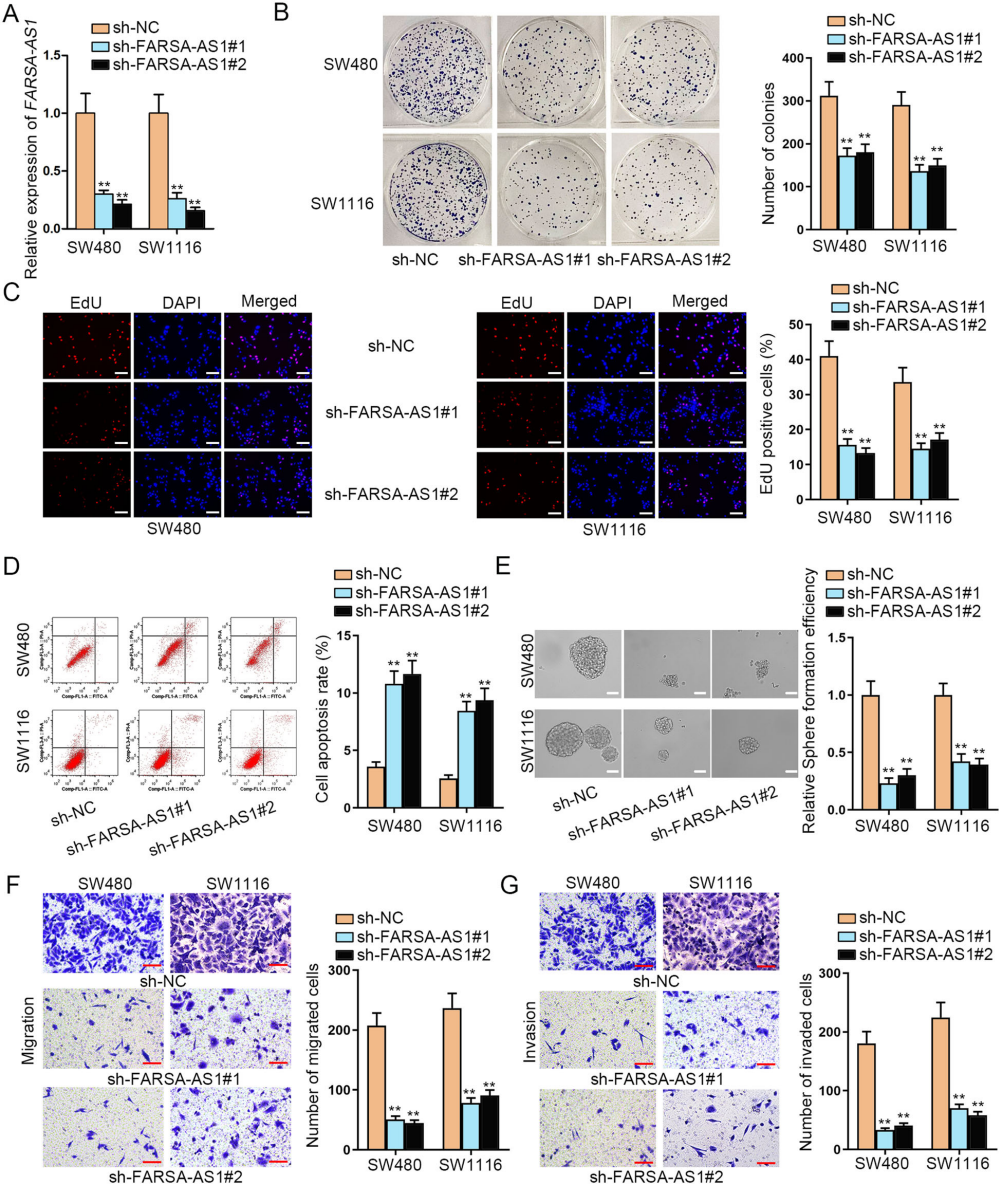
*FARSA-AS1* facilitates CRC cell growth, stemness, migration, and invasion. **A** The silencing efficiency of *FARSA-AS1* was tested through qRT-PCR. **B, C** Cell proliferation ability was tested by colony formation assay and EdU assay (scale bar = 150 μm). **D** Flow cytometry analysis was conducted for detecting cell apoptosis. **E** Tumor sphere-formation assay (scale bar = 75 μm) was utilized to detect cell stemness. **F, G** Transwell assay was carried out to test cell migration and invasion capabilities (scale bar = 250 μm). **P < 0.01.

To explore the role of *FARSA-AS1* in CRC, we stably silenced *FARSA-AS1* expression in SW480 and SW1116 cells by transfecting with sh-FARSA-AS1#1/2 (Fig. 3A). As a result, silencing *FARSA-AS1* also led to declined activity of Wnt/β-catenin pathway in both CRC cells (Fig. S2C). Moreover, *FARSA-AS1* depletion remarkably suppressed the proliferation of SW480 and SW1116 cells (Fig. 3B, C). Flow cytometry elucidated the promoted apoptosis in *FARSA-AS1* downregulated SW480 and SW1116 cells (Fig. 3D). Importantly, knockdown of *FARSA-AS1* markedly repressed the ability of SW480 and SW1116 cells to form primary, secondary, and tertiary spheres (Fig. 3E and Fig. S2D). Also, the levels of *ALDH* and *CD133* were reduced in CRC cells in response to *FARSA-AS1* absence (Fig. S2E). Similarly, the inhibited migration and invasion were observed in SW480 and SW1116 cells with *FARSA-AS1* deficiency (Fig. 3F, G). In sum, *FARSA-AS1* facilitates CRC cell growth, stemness, migration, and invasion.

### FARSA-AS1 upregulates SOX9 by sponging miR-18b-5p

**Fig. 4:**
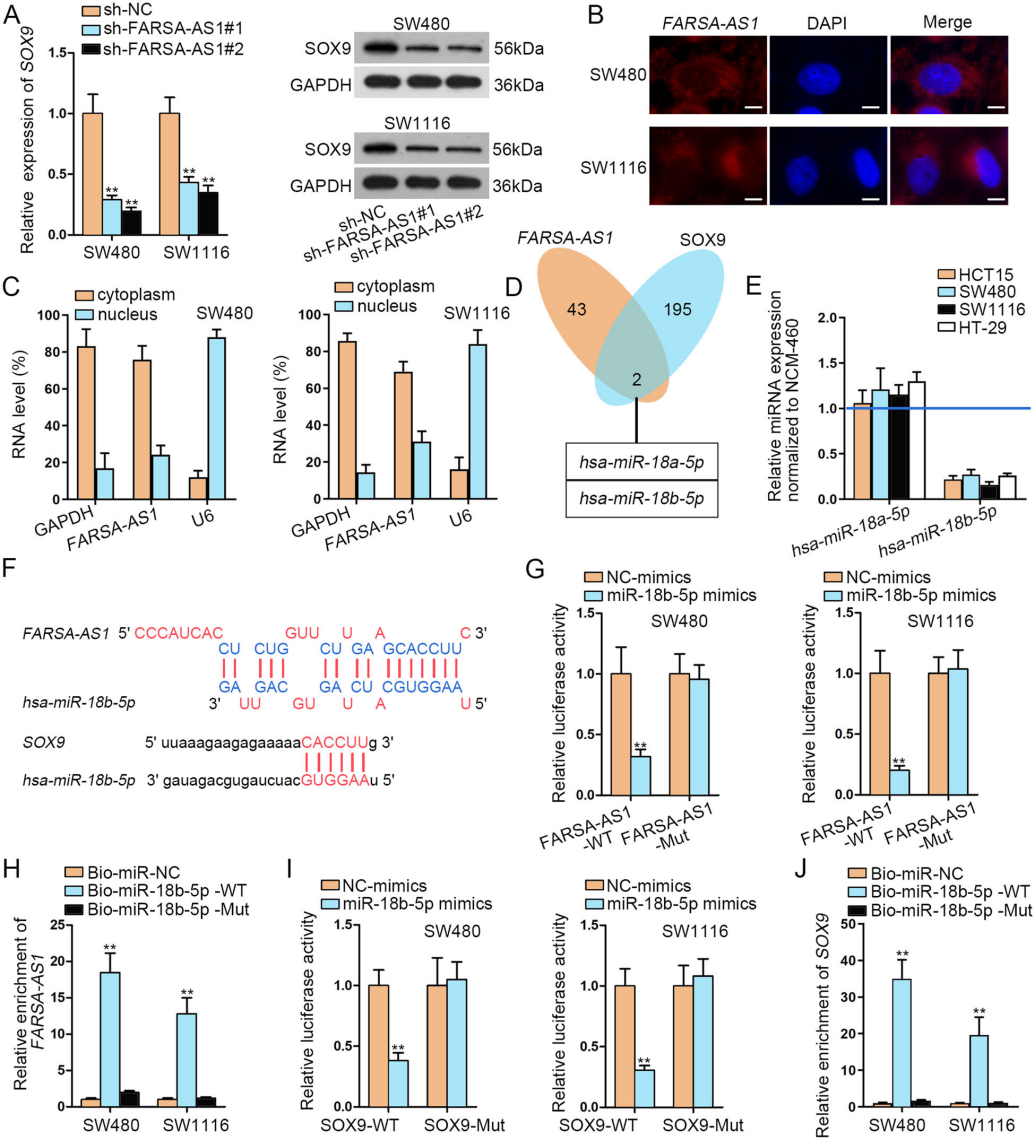
*FARSA-AS1* upregulates *SOX9* by sponging *miR-18b-5p*. **A** The effect of silencing *FARSA-AS1* on *SOX9* expression was tested by qRT-PCR and western blot experiments. **B, C** FISH assay (scale bar = 10 μm) and subcellular fractionation assay were conducted to test the location of *FARSA-AS1* in SW480 and SW1116 cells. **D** Two miRNAs shared by *FARSA-AS1* and *SOX9* were picked out through DIANA and starBase. **E** The expressions of *miR-18a-5p* and *miR-18b-5p* were detected in CRC cells by qRT-PCR. **F** The binding sites between *miR-18b-5p* and *FARSA-AS1*/*SOX9*. **G, H** Luciferase reporter and RNA pull-down experiments were adopted to prove the binding between *miR-18b-5p* and *FARSA-AS1*. **I, J** The interaction between *miR-18b-5p* and *SOX9* was detected through luciferase reporter and RNA pull-down experiments. **P < 0.01.

Interestingly, we then discovered that the levels of SOX9 mRNA and protein were both reduced along with *FARSA-AS1* downregulation in SW480 and SW1116 cells (Fig. 4A). To explore the mechanism of *FARSA-AS1* in regulating *SOX9*, we tested the localization of *FARSA-AS1* in SW480 and SW1116 cells via FISH and subcellular fractionation assays. The results demonstrated that *FARSA-AS1* was mainly distributed in the cytoplasm of both CRC cells (Fig. 4B, C), which suggested the regulation of *FARSA-AS1* on *SOX9* at post-transcriptional level. It has been reported that lncRNAs could regulate the expression of mRNAs by functioning as a ‘miRNA Sponge’^19,27^. Later, we aimed to find out the potential miRNAs that could simultaneously bind to *FARSA-AS1* and *SOX9*. In this case, 45 miRNAs were predicted for *FARSA-AS1* through DIANA (http://diana.imis.athena-innovation.gr/DianaTools/index.php?r=site/page&view=software), and 197 miRNAs were predicted for *SOX9* via starBase (http://starbase.sysu.edu.cn/index.php), with only two miRNAs (*miR-18a-5p* and *miR-18b-5p*) shared between them (Fig. 4D). Further, only *miR-18b-5p* was validated to be under-expressed in CRC cells in comparison to NCM-460 cells (Fig. 4E). Then, the binding sequences between *miR-18b-5p* and *FARSA-AS1* or *SOX9* were shown (Fig. 4F). Before verifying the interaction between them, we ascertained that *miR-18b-5p* expression was increased in CRC cells after transfecting with miR-18b-5p mimics (Fig. S2F). As validated by the data from luciferase reporter assay, enhanced expression of *miR-18b-5p* lessened the luciferase activity of FARSA-AS1-WT but not that of FARSA-AS1-Mut (Fig. 4G). In addition, RNA pull-down assay results confirmed the specific interaction of *FARSA-AS1* with miR-18b-5p-WT rather than miR-18b-5p-Mut (Fig. 4H). Meanwhile, the combination between *miR-18b-5p* and *SOX9* was testified similarly by such manners (Fig. 4I, J). Taken all together, *FARSA-AS1* upregulates *SOX9* in CRC by sponging *miR-18b-5p*.

### SOX9 fully rescues the effects of FARSA-AS1 on CRC cells

**Fig. 5:**
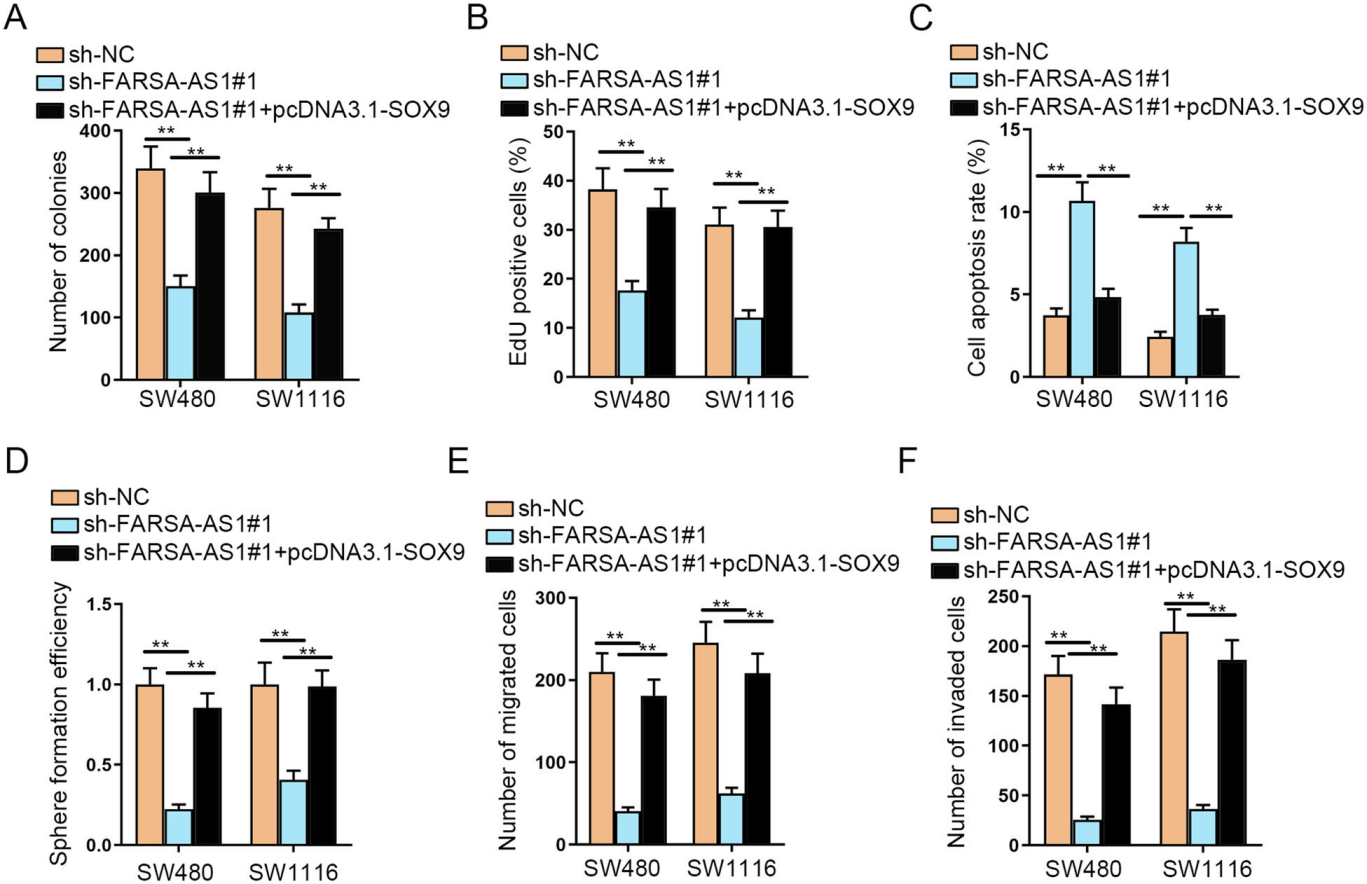
*SOX9* fully rescues the effects of *FARSA-AS1* on CRC cells. **A, B** Cell proliferation was tested by colony formation and EdU experiments. **C** Flow cytometry experiment was carried out to test cell apoptosis. **D** Tumor sphere-formation assay was carried out for detecting cell stemness. **E, F** The migration and invasion capabilities of cells were estimated via Transwell assay. **P < 0.01.

To the purpose of verifying whether the regulation of *FARSA-AS1* on CRC progression was mediated by *SOX9*, some restoration assays were carried out. According to the outcomes of colony formation and EdU assays, we found that *SOX9* overexpression fully rescued the inhibitory effect of *FARSA-AS1* silencing on CRC cell proliferation (Fig. 5A, B and Fig. S3A, B). The induced apoptosis in *FARSA-AS1* depleted cells was also fully recovered by upregulated *SOX9* (Fig. 5C and Fig. S3C). *FARSA-AS1* knockdown inhibited the sphere-formation efficiency of SW480 and SW1116 cells, whereas the cotransfection of pcDNA3.1-SOX9 fully counteracted this effect (Fig. 5D and Fig. S3D). Meanwhile, the suppressed ability of CRC cells to form secondary and tertiary spheres was completely recovered due to *SOX9* upregulation (Fig. S3E), so were the restrained levels of *ALDH* and *CD133* (Fig. S3F). In addition, ectopic expression of *SOX9* fully countervailed *FARSA-AS1* deficiency-mediated suppression on SW480 and SW1116 cell migration and invasion (Fig. 5E, F and Fig. S3G, H). All the results indicated that *SOX9* fully rescues the effects of *FARSA-AS1* on the phenotypes of CRC cells.

### FARSA-AS1 upregulates FARSA via sequestering miR-28-5p

**Fig. 6:**
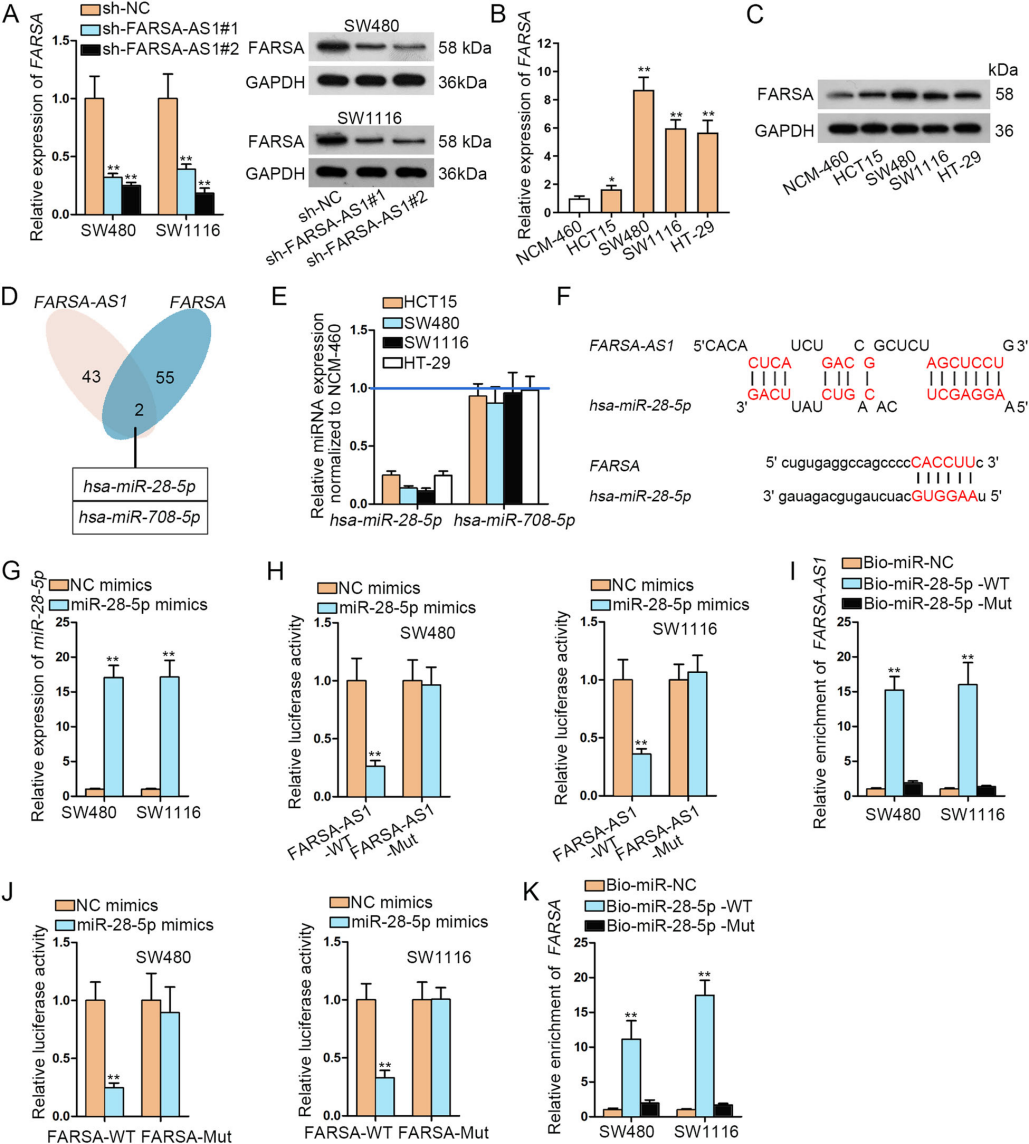
*FARSA-AS1* upregulates *FARSA* via sequestering *miR-28-5p*. **A** The expression of *FARSA* was tested by qRT-PCR and western blot analyses in CRC cells after knocking down *FARSA-AS1*. **B, C** The qRT-PCR and western blot were adopted to detect the expression of *FARSA* in CRC cells. **D** Two shared miRNAs of *FARSA-AS1* and *FARSA* were found by DIANA and starBase. **E** The expression of *miR-28-5p* and *miR-708-5p* in CRC cells was detected by qRT-PCR. **F** The binding sequence between *miR-28-5p* and *FARSA-AS1* and that between *FARSA* and *miR-28-5p* were shown. **G** The overexpression efficiency of *miR-28-5p* was tested by qRT-PCR. **H**, **I** The experiments of luciferase reporter and RNA pull-down were conducted to confirm the binding between *miR-28-5p* and *FARSA-AS1*. **J, K** The combination of *miR-28-5p* with *FARSA* was confirmed through luciferase reporter experiment and RNA pull-down assay. *P < 0.05, **P < 0.01.

Through UCSC (http://genome.ucsc.edu/), *FARSA*, the nearby gene of *FARSA-AS1*, was found. It has been reported that lncRNAs could exert functions in cancer by regulating their nearby genes^28^. Therefore, we wondered the influence of *FARSA-AS1* on *FARSA* expression in CRC cells. As illustrated in Fig. 6A, FARSA mRNA and protein expressions were significantly decreased upon *FARSA-AS1* knockdown. Besides, we found that FARSA mRNA and protein levels were highly expressed in CRC cells in contrast to normal NCM-460 cells (Fig. 6B, C). Interestingly, luciferase reporter assay results displayed that the luciferase activity of *FARSA* 3’UTR presented no evident differences in cells with *miR-18b-5p* upregulation (Fig. S4A). In addition, *miR-18b-5p* overexpression did not affect FARSA mRNA and protein levels in both CRC cells (Fig. S4B). These data revealed that *FARSA-AS1* regulated *FARSA* expression not by sponging *miR-18b-5p*. In consequence, we searched for miRNAs that combined with *FARSA-AS1* and *FARSA*, and then *miR-28-5p* and *miR-708-5p* were screened out (Fig. 6D). Furthermore, the results of qRT-PCR manifested that *miR-28-5p* was downregulated in CRC cells, while *miR-708-5p* expression showed no notable changes (Fig. 6E). Hence, we speculated that *miR-28-5p* might be implicated in the regulation of *FARSA-AS1* on *FARSA* in CRC. The predicted binding sequences between *miR-28-5p* and *FARSA-AS1* or *FARSA* were illustrated in Fig. 6F. Then, we overexpressed *miR-28-5p* in SW480 and SW1116 cells (Fig. 6G). As anticipated, the luciferase activity of FARSA-AS1-WT was considerably diminished with the transfection of miR-28-5p mimics, while *miR-28-5p* overexpression did not impact the luciferase activity of FARSA-AS1-Mut (Fig. 6H). RNA pull-down assay data implied the remarkable enrichment of *FARSA-AS1* specially by biotinylated miR-28-5p-WT (Fig. 6I). Furthermore, the interaction between *miR-28-5p* and *FARSA* was also validated by luciferase reporter assay and RNA pull-down assay (Fig. 6J, K). All data suggested that *FARSA-AS1* upregulates *FARSA* via sequestering *miR-28-5p*.

### FARSA-AS1 enhances SOX9 and FARSA expressions to promote tumor progression in CRC

**Fig. 7:**
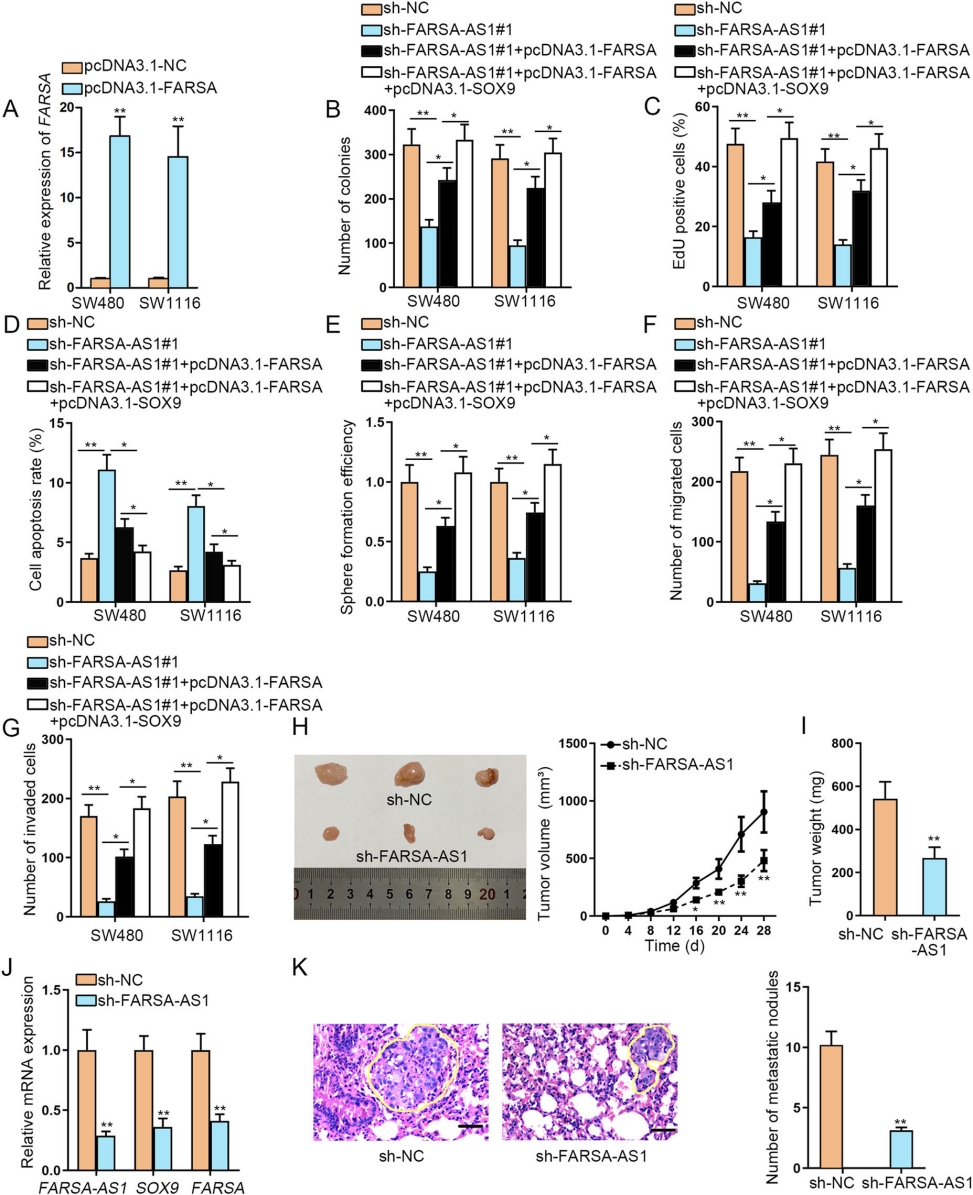
*FARSA-AS1* enhances CRC progression by upregulating *SOX9* and *FARSA*. **A** The qRT-PCR was conducted to test the overexpression efficiency of *FARSA* in SW480 and SW1116 cells. **B, C** The capability of cell proliferation was tested by EdU experiment and colony formation experiment. **D** Flow cytometry experiment was carried out to test cell apoptosis. **E** Tumor sphere-formation assay was carried out for detecting cell stemness. **F, G** The capability of cell migration and invasion was estimated via Transwell assay. **H** The representative pictures and the growth curves of tumors derived from sh-NC- or sh-FARSA-AS1-transfected CRC cells. **I** Tumor weight was estimated in above two groups. **J** The expressions of *FARSA-AS1*, *SOX9*, and *FARSA* were detected separately by qRT-PCR in tumors with or without silenced *FARSA-AS1*. **K** The representative images of HE staining (scale bar = 150 μm) of lung from indicated groups, and the quantification bar graph of lung metastatic nodules in tumors with or without silenced *FARSA-AS1* were shown. *P < 0.05, **P < 0.01.

To determine whether *FARSA-AS1* functions in CRC by targeting *SOX9* and *FARSA*, a series of rescue experiments were designed and conducted. At first, we tested the overexpression efficiency of pcDNA3.1-FARSA in CRC cells, and results illustrated that *FARSA* expression was remarkably increased by pcDNA3.1-FARSA (Fig. 7A). As a result, overexpressed *FARSA* partially rescued the suppressed proliferation in *FARSA-AS1* depleted cells, whereas the cotransfection of pcDNA3.1-FARSA and pcDNA3.1-SOX9 countervailed the repressive effect of *FARSA-AS1* silencing on cell proliferation (Fig. 7B, C and Fig. S4C, D). *FARSA-AS1* knockdown promoted the apoptosis of CRC cells, but this impact was partially recovered by *FARSA* upregulation, and mostly offset with the cotransfection of pcDNA3.1-FARSA and pcDNA3.1-SOX9 (Fig. 7D and Fig. S4E). The hampered stemness of *FARSA-AS1* silenced SW480 and SW1116 cells was partially reversed by transfecting with pcDNA3.1-FARSA, while restored upon the co-effect of *FARSA* overexpression and *SOX9* upregulation (Fig. 7E and Fig. S4F, G). Also, the alleviated cell migration and invasion caused by silenced *FARSA-AS1* was partially recovered by pcDNA3.1-FARSA transfection, but normalized in response to the co-upregulation of *FARSA* and *SOX9* (Fig. 7F, G and Fig. S4H). In the meantime, we also conducted in vivo assays to further verify the importance of *FARSA-AS1* for CRC progression. As expected, the tumors from *FARSA-AS1*-silenced CRC cells looked smaller owing to the slower growth rate compared with those from sh-NC group (Fig. 7H). Resultantly, the weight of tumors in sh-FARSA-AS1 group was also lighter than that in control group (Fig. 7I). Further, we certified the reduced levels of *FARSA-AS1*, *SOX9* and *FARSA* in tumors derived from CRC cells with silenced *FARSA-AS1* (Fig. 7J). Moreover, the results of in vivo tumor metastasis model showed that the number of lung metastatic nodules was remarkably decreased in mice injected with *FARSA-AS1*-silenced CRC cells (Fig. 7K). In conclusion, *FARSA-AS1* contributes to CRC progression by upregulating *SOX9* and *FARSA*.

## Discussion

As a common malignancy, the incidence and mortality of CRC are increasing worldwide^29^. In spite of the research over years, we have not fully elucidated the mechanism underlying CRC tumorigenesis. Until now, we have not identified the effective diagnostic methods. Most patients with CRC are diagnosed at the middle or advanced stage and miss the chance to be timely treated^30^. Therefore, it is in an urgent need to explore the potential biomarkers for the treatment of CRC. Here, we searched TGCA database and found a *SOX9* presented a high level in CRC tissues. In various cancer types, *SOX9* has been observed to be deregulated because of the amplification and other mechanisms^31,32^. Recent researches showed that the upregulation of *SOX9* predicted unsatisfactory survival rate in hepatocellular carcinoma^33^ and gastric cancer^6^. In our study, the results demonstrated that *SOX9* was remarkably upregulated in CRC cells, and *SOX9* knockdown suppressed cell proliferation, stemness, migration, and invasion. SOX9 was also reported as a transcription factor to activate the expression of some genes^34^. Here, we found that silencing *SOX9* decreased the expression of lncRNA *FARSA-AS1*, and *SOX9* overexpression increased *FARSA-AS1* expression. In addition, SOX9 was also confirmed to combine with FARSA-AS1 promoter. These data manifested that SOX9 is an oncogene in CRC and functions as a transcription activator of *FARSA-AS1* in CRC cells.

In recent years, the critical effects of lncRNAs have attracted the attentions of many researchers for the reason of their abnormal regulation on tumor progression. In particular, an increasing number of lncRNAs have been identified as biomarkers in cancers^35^. Although a series of lncRNAs have been found to be dysregulated in multiple cancers, it is still needed to investigate the molecular mechanism of lncRNAs in cancers. As is widely reported, lncRNAs are associated with the progression of diverse cancers, including CRC. For example, *LINC01296* contributes to CRC progression via PI3K/AKT pathway and targeting *miR-26a*/*GALNT3* axis^36^. YAP1-mediated *MALAT1* enhances CRC angiogenesis and epithelial-mesenchymal transition via blocking *miR-126-5p*^37^. *FARSA-AS1* is a newly identified lncRNA, which has not been researched in CRC. In this study, *FARSA-AS1* was found to be highly expressed in CRC cells. Silencing *FARSA-AS1* inhibited cell proliferation, stemness, migration, and invasion. All results suggested that *FARSA-AS1* presented a carcinogenic property in CRC.

MicroRNAs (miRNAs) are identified as small noncoding RNAs with about 22 nucleotides in length. MiRNAs have been revealed to play important parts in regulating the biological behaviors of cancer cells. For instance, *miR-20*0c/141 targets to HIPK1/β-catenin axis to regulate the heterogeneity of breast cancer stem cells^38^. *MiR-4516* functions as a novel oncogene in glioblastoma and predicts poor prognosis via targeting *PTPN14*^39^. *MiR-371a-3p* targets to *TOB1* and facilitates cell proliferation and invasion in gastric cancer^40^. Generally, lncRNAs regulate the expression of mRNAs by secluding miRNAs. Therefore, miRNAs are of great importance in ceRNA network^41^. *MiR-18b-5p* was reported in lung adenocarcinoma^42^, whereas its role and molecular mechanism remain largely unclear in CRC. In this study, we found that *FARSA-AS1* regulated *SOX9* by sponging *miR-18b-5p*. Restoration assay manifested that *SOX9* upregulation fully rescued the inhibitive effects of *FARSA-AS1* silencing on the functions of CRC cells. Then, we found that *FARSA-AS1* could also modulate its nearby gene, *FARSA*, through sponging *miR-28-5p*. According to the results of rescue experiments, the impacts of *FARSA-AS1* downregulated cells were partially counteracted by *FARSA* upregulation, but mostly recovered upon the co-overexpression of *FARSA* and *SOX9*.

In conclusion, *FARSA-AS1* was upregulated in CRC cells and facilitated CRC progression by upregulating *SOX9* and *FARSA*. This discovery provides a helpful theoretical basis for the exploration of effective CRC therapeutic strategies.

## Supplementary information

Figure S1

Figure S2

Figure S3

Figure S4

Supplementary information

## References

[1] Booth RA (2007). Minimally invasive biomarkers for detection and staging of colorectal cancer. Cancer Lett..

[2] Jemal A (2011). Global cancer statistics. CA Cancer J. Clin..

[3] Arends MJ (2013). Pathways of colorectal carcinogenesis. Appl. Immunohistochem. Mol. Morphol..

[4] Foster JW (1994). Campomelic dysplasia and autosomal sex reversal caused by mutations in an SRY-related gene. Nature.

[5] Wagner T (1994). Autosomal sex reversal and campomelic dysplasia are caused by mutations in and around the SRY-related gene SOX9. Cell.

[6] Santos JC (2016). SOX9 elevation acts with canonical WNT signaling to drive gastric cancer progression. Cancer Res..

[7] Ma F (2016). SOX9 drives WNT pathway activation in prostate cancer. J. Clin. Invest..

[8] Kawai T (2016). SOX9 is a novel cancer stem cell marker surrogated by osteopontin in human hepatocellular carcinoma. Sci. Rep..

[9] Richtig G (2017). SOX9 is a proliferation and stem cell factor in hepatocellular carcinoma and possess widespread prognostic significance in different cancer types. PLoS ONE.

[10] Huang J, Guo L (2017). Knockdown of SOX9 inhibits the proliferation, invasion, and EMT in thyroid cancer cells. Oncol. Res..

[11] Liu C (2016). Sox9 regulates self-renewal and tumorigenicity by promoting symmetrical cell division of cancer stem cells in hepatocellular carcinoma. Hepatology.

[12] Gutschner T, Diederichs S (2012). The hallmarks of cancer: a long non-coding RNA point of view. RNA Biol..

[13] Saxena A, Carninci P (2011). Long non-coding RNA modifies chromatin: epigenetic silencing by long non-coding RNAs. Bioessays.

[14] Wang KC, Chang HY (2011). Molecular mechanisms of long noncoding RNAs. Mol. Cell.

[15] Peng W, Wang Z, Fan H (2017). LncRNA NEAT1 impacts cell proliferation and apoptosis of colorectal cancer via regulation of Akt signaling. Pathol. Oncol. Res..

[16] Wang Y (2017). LncRNA AB073614 regulates proliferation and metastasis of colorectal cancer cells via the PI3K/AKT signaling pathway. Biomed. Pharmacother..

[17] Li C, Gao Y, Li Y, Ding D (2017). TUG1 mediates methotrexate resistance in colorectal cancer via miR-186/CPEB2 axis. Biochem. Biophys. Res. Commun..

[18] Cesana M (2011). A long noncoding RNA controls muscle differentiation by functioning as a competing endogenous RNA. Cell.

[19] Tay Y, Rinn J, Pandolfi PP (2014). The multilayered complexity of ceRNA crosstalk and competition. Nature.

[20] Li H (2017). Long noncoding RNA NORAD, a novel competing endogenous RNA, enhances the hypoxia-induced epithelial-mesenchymal transition to promote metastasis in pancreatic cancer. Mol. Cancer.

[21] Lv M (2017). lncRNA H19 regulates epithelial-mesenchymal transition and metastasis of bladder cancer by miR-29b-3p as competing endogenous RNA. Biochim. Biophys. Acta Mol. Cell Res..

[22] Huang W (2017). The long non-coding RNA SNHG3 functions as a competing endogenous RNA to promote malignant development of colorectal cancer. Oncol. Rep..

[23] Serrano-Oviedo L (2020). Identification of a stemness-related gene panel associated with BET inhibition in triple negative breast cancer. Cell Oncol. (Dordr.).

[24] Zheng A (2019). Long non-coding RNA LUCAT1/miR-5582-3p/TCF7L2 axis regulates breast cancer stemness via Wnt/β-catenin pathway. J. Exp. Clin. Cancer Res..

[25] Prévostel C (2016). SOX9 is an atypical intestinal tumor suppressor controlling the oncogenic Wnt/ß-catenin signaling. Oncotarget.

[26] Wang L (2019). SOX9/miR-203a axis drives PI3K/AKT signaling to promote esophageal cancer progression. Cancer Lett..

[27] Karreth FA, Pandolfi P (2013). P. ceRNA cross-talk in cancer: when ce-bling rivalries go awry. Cancer Discov..

[28] Qian W (2019). lncRNA ZEB1-AS1 promotes pulmonary fibrosis through ZEB1-mediated epithelial-mesenchymal transition by competitively binding miR-141-3p. Cell Death Dis..

[29] Siegel RL, Miller KD, Jemal A (2018). Cancer statistics, 2018. CA Cancer J. Clin..

[30] Yu J (2017). Metagenomic analysis of faecal microbiome as a tool towards targeted non-invasive biomarkers for colorectal cancer. Gut.

[31] Wang L (2017). Linc-ROR promotes esophageal squamous cell carcinoma progression through the derepression of SOX9.. J. Exp. Clin. Cancer Res..

[32] Wang L (2019). Unbalanced YAP-SOX9 circuit drives stemness and malignant progression in esophageal squamous cell carcinoma. Oncogene.

[33] Leung CO (2016). Sox9 confers stemness properties in hepatocellular carcinoma through Frizzled-7 mediated Wnt/beta-catenin signaling. Oncotarget.

[34] Gubbay J (1990). A gene mapping to the sex-determining region of the mouse Y chromosome is a member of a novel family of embryonically expressed genes. Nature.

[35] Chandra Gupta S, Nandan Tripathi Y (2017). Potential of long non-coding RNAs in cancer patients: From biomarkers to therapeutic targets. Int. J. Cancer.

[36] Liu B (2019). Correction to: LINC01296/miR-26a/GALNT3 axis contributes to colorectal cancer progression by regulating O-glycosylated MUC1 via PI3K/AKT pathway. J. Exp. Clin. Cancer Res..

[37] Sun Z (2019). YAP1-induced MALAT1 promotes epithelial-mesenchymal transition and angiogenesis by sponging miR-126-5p in colorectal cancer. Oncogene.

[38] Liu B (2018). miR-200c/141 regulates breast cancer stem cell heterogeneity via targeting HIPK1/beta-catenin axis. Theranostics.

[39] Cui T (2019). miR-4516 predicts poor prognosis and functions as a novel oncogene via targeting PTPN14 in human glioblastoma. Oncogene.

[40] Guo H (2019). MicroRNA-371a-3p promotes progression of gastric cancer by targeting TOB1. Cancer Lett..

[41] Tan JY (2015). Extensive microRNA-mediated crosstalk between lncRNAs and mRNAs in mouse embryonic stem cells. Genome Res..

[42] Xue, M. et al. lncRNA ZFPM2-AS1 promotes proliferation via miR-18b-5p/VMA21 axis in lung adenocarcinoma. *J. Cell. Biochem.* **121**, 313–321 (2019). 10.1002/jcb.29176 31297866

