# Supplementary figures and images for: SOX9-activated FARSA-AS1 predetermines cell growth, stemness, and metastasis in colorectal cancer through upregulating FARSA and SOX9
Source: Cell Death Dis. 2020 Dec 14;11(12):1071. doi: 10.1038/s41419-020-03273-4 (PMC7736271; doi:10.1038/s41419-020-03273-4)

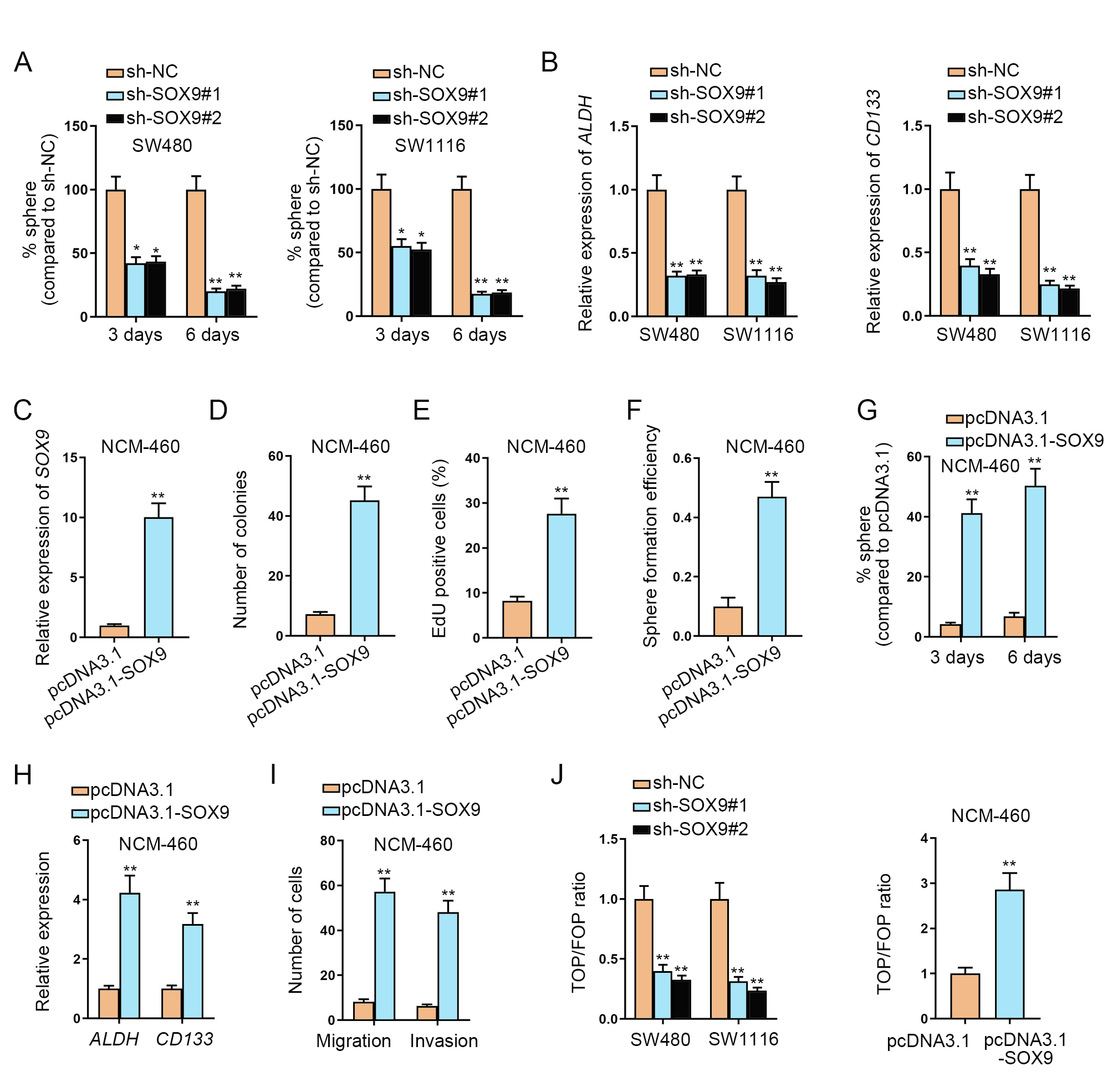

Supplement: Supplementary file 1 — Figure S1 [file 41419_2020_3273_MOESM1_ESM.tif]

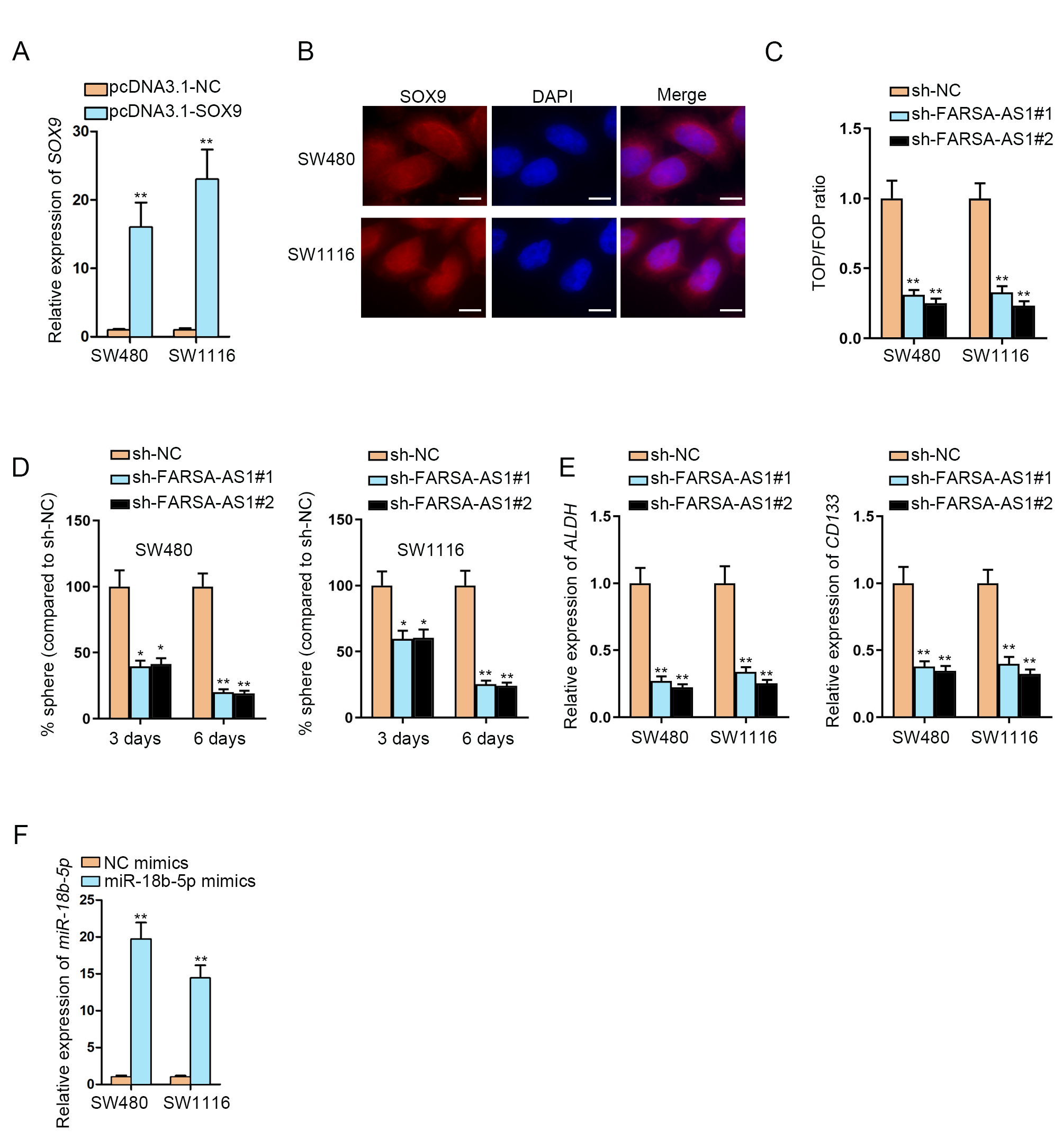

Supplement: Supplementary file 2 — Figure S2 [file 41419_2020_3273_MOESM2_ESM.tif]

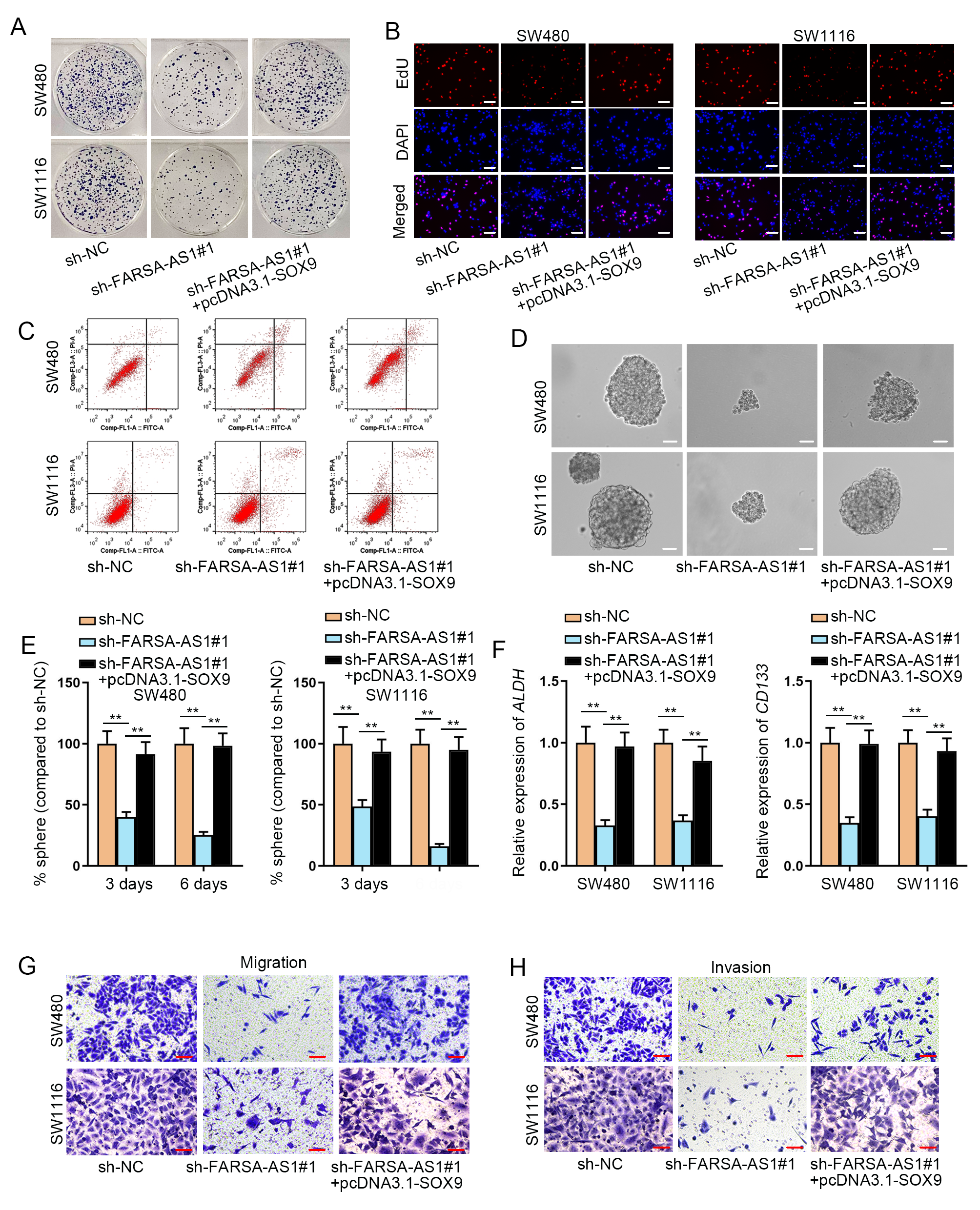

Supplement: Supplementary file 3 — Figure S3 [file 41419_2020_3273_MOESM3_ESM.tif]

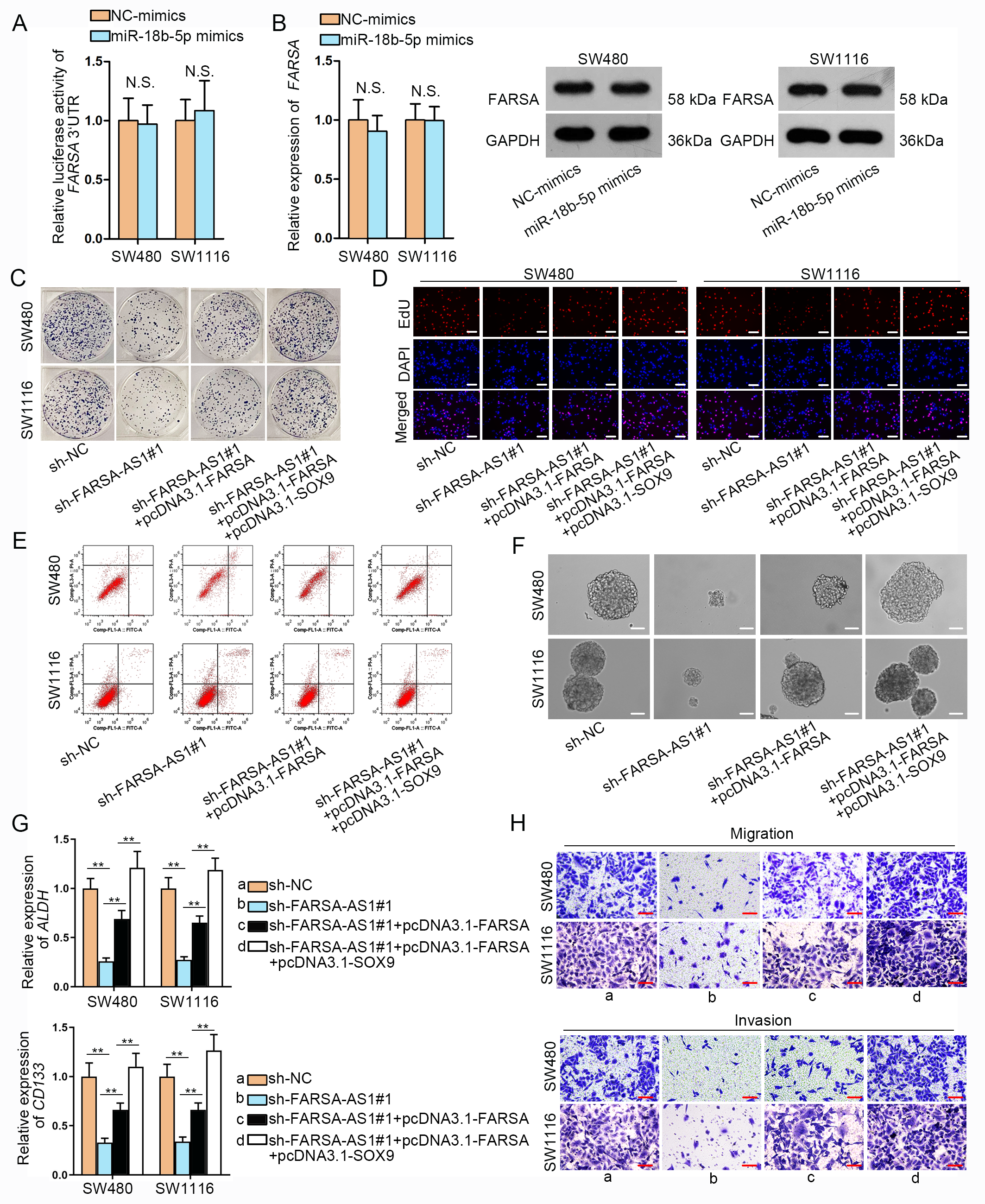

Supplement: Supplementary file 4 — Figure S4 [file 41419_2020_3273_MOESM4_ESM.tif]
