# Supplementary material for: SOX9-activated FARSA-AS1 predetermines cell growth, stemness, and metastasis in colorectal cancer through upregulating FARSA and SOX9
Source: Cell Death Dis. 2020 Dec 14;11(12):1071. doi: 10.1038/s41419-020-03273-4 (PMC7736271; doi:10.1038/s41419-020-03273-4)
Supplement: Supplementary file 5 — Supplementary information [file 41419_2020_3273_MOESM5_ESM.doc]

**Figure S1**

(A) Secondary and tertiary tumor sphere formation assays were further performed to detect changes in cell stemness. (B) The levels of two stemness-related markers including *ALDH* and *CD133* were evaluated by qRT-PCR. (C) The overexpression efficiency of *SOX9* in NCM-460 cells was confirmed via qRT-PCR. (D-E) Colony formation and EdU assays examined the proliferation of NCM-460 cells before and after *SOC9* upregulation. (F-G) Primary, secondary and tertiary tumor sphere formation assays tested the stemness of NCM-460 cells with or without *SOX9* elevation. (H) The levels of *ALDH* and *CD133* were tested via qRT-PCR. (I) Transwell assay detected the migration and invasion of indicated NCM-460 cells. (J) TOP/FOP flash assay detected the impact of *SOX9* inhibition or overexpression on the activity of Wnt/β-catenin pathway. ^**^P < 0.01.

**Figure S2**

(A) The overexpression efficiency of *SOX9* in SW480 and SW1116 cells was validated by qRT-PCR. (B) The location of SOX9 protein in above two CRC cells was estimated via IF staining. (C) TOP/FOP flash assay detected the impact of *FARSA-AS1* depletion on the activity of Wnt/β-catenin pathway. (D) Secondary and tertiary tumor sphere formation assays were further performed to detect changes in cell stemness. (E) The levels of *ALDH* and *CD133* were tested via qRT-PCR. (F) The overexpression efficiency of *miR-18b-5p* in two CRC cells was verified via qRT-PCR. ^*^P < 0.05, ^**^P < 0.01.

**Figure S3**

(A-D) The corresponding image data for functional assays conducted in Figure 5A-D. (E) Secondary and tertiary tumor sphere formation assays were further performed to detect changes in cell stemness under diverse conditions. (F) The levels of *ALDH* and *CD133* in indicated cells were tested via qRT-PCR. (G-H) The images of Transwell assays performed in Figure 5E-F. ^**^P < 0.01.

**Figure S4**

(A) The luciferase reporter experiment was adopted to test the binding between *miR-18b-5p* and *FARSA*. (B) The influence of *miR-18b-5p* upregulation on *FARSA* expression was tested by qRT-PCR and western blot experiments. (C-F) The corresponding image data for functional assays conducted in Figure 7B-E. (G) The levels of *ALDH* and *CD133* in indicated cells were tested via qRT-PCR. (H) The images of Transwell assays performed in Figure 7F-G. ^**^P < 0.01. N.S. meant not significant.
